# FAP-Targeted Radionuclide Therapy: Mechanisms, Clinical Applications, and Combination Strategies

**DOI:** 10.3390/biomedicines14071479

**Published:** 2026-06-30

**Authors:** Ayça Arçay Öztürk, Rita Saúde-Conde, Juanito Gebruers, Patrick Flamen

**Affiliations:** 1 Department of Nuclear Medicine, Institute Jules Bordet, Hôpital Universitaire de Bruxelles, 1070 Brussels, Belgium; juanito.gebruers@hubruxelles.be (J.G.);; 2 Department of Digestive Oncology, Institute Jules Bordet, Hôpital Universitaire de Bruxelles, 1070 Brussels, Belgium

**Keywords:** theranostics, fibroblast activation protein, FAPI, FAP, FAPI PET, radionuclide therapy, combination, immunotherapy, targeted radionuclide therapy

## Abstract

The fibroblast activation protein (FAP) has emerged as a compelling theranostic target because it is highly expressed in the tumour microenvironment of many solid malignancies, predominantly on cancer-associated fibroblasts and, in selected tumour types, also on tumour cells. Following the rapid clinical expansion of FAP-targeted PET imaging, FAP-targeted radionuclide therapy (FAP-TRT) is now being explored as a predominantly stromal-directed therapeutic strategy across a broad range of solid malignancies. However, unlike established theranostic paradigms, such as prostate-specific membrane antigen- and somatostatin receptor-directed radioligand therapies, FAP-TRT faces distinct biological and translational challenges, including stromal heterogeneity, variable patterns of FAP expression, and limited tumour retention of many early radioligands. This review outlines the biological rationale, mechanistic basis, radiopharmaceutical development, and emerging clinical evidence for FAP-TRT. We highlight the recent ligand-engineering strategies aimed to improve tumour residence time and absorbed dose, and to summarise the current clinical data with particular focus on dosimetry, safety, and early efficacy signals. We also discuss key future directions, including disease-focused clinical development and rational combination strategies with immune checkpoint inhibitors, DNA damage response inhibitors, and chemotherapy. Overall, the available data support the feasibility of FAP-TRT but also underscore the need for improved ligand design and biologically informed clinical development to define its role within the evolving theranostic landscape.

## 1. Introduction

Cancer-associated fibroblasts (CAFs) are major cellular components of the tumour microenvironment and actively contribute to extracellular matrix remodelling, tumour growth, immune evasion, and therapeutic resistance [1]. Their functional heterogeneity is increasingly recognised, with distinct CAF subsets exerting tumour-promoting, immune-excluding, or, in some contexts, tumour-restraining effects [2]. The fibroblast activation protein (FAP), a type II transmembrane serine protease, is one of the CAF-associated markers and is expressed at low levels in most normal adult tissues but is upregulated in activated stromal fibroblasts and across many solid tumours. This specific physiologic expression pattern, together with its broad presence in tumour-associated stroma, has established the FAP as an attractive target for molecular imaging and therapy in oncology.

Clinical interest in FAP-targeted imaging began with antibody-based approaches in the 1990s, including imaging with the anti-FAP monoclonal antibody F19 [3]. Clinical translation accelerated recently, after the introduction of quinoline-based FAP inhibitors (FAPI) in 2018 [4], which enabled high-contrast PET imaging using small-molecule radiotracers. In particular, the development of FAPI-02, and the subsequent optimisation toward FAPI-04 and FAPI-46, established the foundation for FAP theranostics. These compounds showed a rapid tumour uptake and a favourable tumour-to-background contrast, supporting a broad diagnostic application across multiple malignancies and stimulating therapeutic development [5].

Despite this diagnostic success, therapeutic translation has proven more complex. Many early FAP-targeted ligands showed fast tumour uptake but limited tumour retention, a property that is favourable for imaging but less suitable for sustained radionuclide therapy [6]. In contrast to prostate-specific membrane antigen (PSMA)- and somatostatin receptor (SSTR)-directed radioligand therapies, FAP-targeted radionuclide therapy (FAP-TRT) acts primarily on the stromal compartment rather than exclusively on tumour cells. As a result, treatment efficacy may depend not only on the tracer uptake and dosimetry, but on the tumour architecture, the spatial relationship between CAFs and malignant cells, the contribution of crossfire irradiation, and the biological consequences of stromal irradiation within the tumour microenvironment, including potential bystander effects [7]. These features likely contribute to the heterogeneous efficacy signals seen in early clinical studies.

The preclinical and clinical development of FAP-TRT therefore requires optimisation at several levels. On the radiopharmaceutical side, current strategies aim to improve the tumour residence time and absorbed dose while maintaining acceptable normal-organ exposure. These include multimerization, albumin-binding approaches, peptide-based ligands, and other structure-modifying strategies. On the clinical side, a key unmet need is the refinement of patient selection. FAP PET positivity alone may not be sufficient to predict therapeutic benefit, and future studies will likely require more quantitative imaging-based enrichment approaches, analogous to other established theranostic platforms. In addition, the development of more representative preclinical models that preserve stromal, tumour, and immune interactions will be essential for improving clinical translation.

In this review, we discuss the biologic basis, radiopharmaceutical evolution, current clinical evidence, and future directions of FAP-TRT. Particular emphasis is placed on stromal biology and mechanisms of action, advances in radiopharmaceutical development, emerging clinical efficacy and safety data, and the role of rational combination strategies in shaping the future clinical development of this theranostic approach.

## 2. Mechanisms and Challenges

FAP-TRT operates through multiple mechanisms, including the direct targeting of FAP-expressing CAFs and tumour cells, as well as the crossfire effect, where radiation impacts adjacent cells. Therefore, the therapeutic efficacy of FAP-TRT depends on several key factors.

Firstly, the type of cells expressing the FAP and the tumour architecture play a crucial role. Unlike established tumour cell-targeted TRTs, such as PSMA- and SSTR-directed radioligand therapies, FAP-TRT predominantly targets the tumour stroma. When FAP is only expressed on CAFs, the spatial organisation of CAFs relative to tumour cells, whether they are closely intertwined with tumour cells or located further away, affects the accessibility and therapeutic effect of FAP-targeted radioligands. In this case, the effect of FAP-TRT relies on a combination of crossfire irradiation of neighbouring tumour cells, direct irradiation of CAFs, and secondary changes induced in the TME. On the other hand, when the FAP is also expressed on tumour cells, the direct targeting effect of FAP-TRT may be more pronounced than just the crossfire irradiation of tumour cells.

Furthermore, the specific characteristics of the FAP-targeting radiopharmaceuticals have a significant effect. The type of radionuclide used, its range, and emission properties (e.g., alpha or beta particles) are critical for targeting FAP+ CAFs and for ensuring effective delivery of radiation. The physical half-life of the radionuclide and the retention time of the radioligand within the tumour also play a key role, influencing the duration of therapeutic exposure and ultimately impacting efficacy.

Additionally, the known radioresistance of CAFs poses an important challenge. The impact of radiation on CAFs, the cells that provide structural support and contribute to immune evasion and therapy resistance in many cancers, remains uncertain, particularly regarding its ability to weaken this protective shield. An additional layer of complexity arises from the pronounced heterogeneity of CAF subpopulations within the tumour microenvironment [1,2]. While many CAF subsets promote immune exclusion, fibrosis, and therapeutic resistance, emerging evidence suggests that certain CAF phenotypes may exert tumour-restraining or immune-supportive functions [1,8]. The unclear impact of radiation on the balance of tumour-supportive and tumour-suppressive CAF subpopulations further complicates the optimisation of FAP-TRT.

Moreover, radiation-induced senescence or phenotypic reprogramming of residual CAFs could further alter the balance between tumour-promoting and tumour-restraining stromal signals [9]. Elucidating the subtype-specific effects of FAP-TRT on CAF biology will therefore be essential to optimise the therapeutic design and avoid unintended microenvironmental consequences. Addressing these complexities requires a deeper understanding of the molecular and cellular effects of radioligands on CAFs, tumour cells, and immune cells.

Another challenge is the development of preclinical tumour models that accurately simulate the TME, incorporating FAP+ CAFs, tumour cells, immune cells, and their interactions. While cancer cell lines transduced to overexpress the human FAP are widely utilised models in preclinical evaluations of FAP-targeted radiopharmaceuticals, they lack clinical relevance, as FAP expression in patients is primarily localised to CAFs within the tumour stroma. According to van der Heide et al., among the available models, U-87 MG cells hold promise for FAP-TRT research, as they recruit murine fibroblasts in vivo, creating heterogeneous FAP expression [10]. To achieve more accurate in vitro evaluations of FAP-TRT efficacy, advanced systems, such as co-cultures and three-dimensional organoid models, are required. Overcoming these obstacles is essential for improving the therapeutic outcomes of FAP-TRT.

## 3. Advances in FAP-Targeted Radiopharmaceutical Development

The clinical exploration of radiopharmaceuticals targeting the FAP began in 1994, with a pivotal study by Welt et al., who performed planar and SPECT imaging using an I-131-labelled monoclonal antibody, mAb F19 [3]. This early groundwork introduced the concept of utilising radiolabelled antibodies to study the biodistribution of the FAP, demonstrating the feasibility of targeting this protein in vivo. A groundbreaking achievement came in 2018 with the development of Gallium-68-labelled FAP inhibitors for PET imaging, which marked a turning point in diagnostic FAP-targeted radiopharmaceuticals. This initial development included two radiotracers: FAPI-01, a radioiodine-labelled compound, and FAPI-02, a precursor capable of chelating radiometals [11]. FAPI-02 showed high tumour specificity but exhibited decreasing uptake over time. To overcome this challenge, the same group in Heidelberg developed a series of piperazine-based FAP inhibitors. Among these, FAPI-04 was identified as the most promising tracer for clinical applications [12] and [^68^Ga]Ga-FAPI-04 remains the most widely used diagnostic FAPI tracer in the literature currently. To further enhance therapeutic efficacy by increasing tumour retention time, the Heidelberg group designed 15 additional FAPI variants. Among these, FAPI-46 demonstrated superior potential as a theranostic agent [13]. However, the tumour retention time of FAPI-46 still remained lower than the approved therapeutic radioligands [^177^Lu]Lu-PSMA-617 and [^177^Lu]Lu-DOTATATE, which exhibit tumour effective half-lives of 54–74 h and 99 h, respectively [14,15] (Table 1).

For TRT, an optimal target and radioligand should provide high tumour specificity, stable and accessible target expression, sufficient tumour retention, favourable tumour-to-normal tissue dose ratios, and effective delivery of absorbed dose to tumour lesions. In theranostic applications, imaging uptake should ideally correlate with the therapeutic dose delivery; however, high uptake on FAP-targeted PET does not necessarily translate into therapeutic efficacy if the tumour retention and tumour absorbed dose remain insufficient [24]. While small molecules have proven highly effective in diagnostic applications, their retention times are inadequate for achieving sustained therapeutic effects. This highlights the main challenge in FAP-targeted radiopharmaceuticals for therapeutic use: enhancing tumour retention while minimising non-specific uptake. To address these limitations, four main strategies are currently under exploration:

- Molecular clustering: e.g., BiOncoFAP [25], DOTAGA.(SA.FAPi)_2_ [21], DOTAGA.Glu.(FAPi)_2_ [26], LNC1013 [27], DOTA-2P(FAPI)_2_ [28] (multimerization of the FAP ligands).
- Extended circulation techniques: e.g., EB-FAPI-B1 [29], EB-FAPI (LNC1004) [20] (by albumin binding).

These two strategies share similar characteristics, notably enhanced tumour retention. The tumour effective half-lives of [^177^Lu]Lu-DOTAGA.(SA.FAPi)_2_ and [^177^Lu]Lu-LNC1004 were reported as 86.6 h and 92 h, respectively, for all lesions [21] [20], surpassing that of [^177^Lu]Lu-PSMA-617 (54 h) [14] (Table 1). However, this improvement in tumour retention is accompanied by increased non-specific uptake in non-target tissues, such as the kidneys and bone marrow, raising concerns about potential toxicity in these two strategies.

C. Target molecule modification: e.g., FAP-2286 [19] and 3BP3940 [30] (replacing quinoline derivatives with peptides).

While [^177^Lu]Lu-FAP-2286 offers improved tumour retention time over FAPI-46, its tumour effective half-life (44 h) [19] is shorter than that of [^177^Lu]Lu-PSMA-617 (54–74 h) and [^177^Lu]Lu-DOTATATE (99 h) [14,15]. Thus, there is still a need for further improvement of tumour retention compared to the approved PSMA and SSTR theranostic ligands. Moreover, in the kidney, [^177^Lu]Lu-FAP-2286 demonstrates a significantly longer effective half-life (81 h) [19] compared to [^177^Lu]Lu-PSMA-617 (42 h) and [^177^Lu]Lu-DOTATATE (52 h) [14,17], again pointing to the risks for potential toxicity. Additionally, the tumour absorbed doses and tumour effective half-life remain lower than the aforementioned radioligands [^177^Lu]Lu-DOTAGA.(SA.FAPi)_2_ and [^177^Lu]Lu-FAPI-46-EB (LNC1004) (Table 1), bringing attention to doubts about their efficacy.

D. Covalent binding approaches: e.g., FAPI-mFS [31] and DOTA-FAPI-Maleimide [32].

Covalent binding holds the potential for increasing drug concentration at the target site, making it a valuable approach to improve the efficacy of therapeutic radiopharmaceuticals targeting the FAP, but challenges arise from non-specific interactions with reactive groups in other proteins and the FAP presence in normal tissues. To enhance treatment effectiveness and to reduce toxicity, it is essential to create targeting agents that selectively bind to the FAP, avoiding healthy tissues. Modern covalent engineering strategies may enhance the tumour uptake and retention of radioligands by ensuring selective targeting while avoiding uncontrollable ligation reactivity during circulation or in healthy tissues.

Overall, optimising targeting strategies and minimising off-target binding are crucial for enhancing the efficacy and safety of FAP-targeted radiopharmaceuticals.

## 4. Clinical Evidence, Efficacy, and Safety of FAP-TRT

The current evidence for FAP-TRT is primarily derived from small case series and first-in-human studies across various tumour types, including mainly sarcoma, breast, thyroid, and pancreatic cancers [33,34]. These studies have largely focused on the feasibility, biodistribution, dosimetry, and safety profiles of different FAP-targeted radiopharmaceuticals, such as [^177^Lu]Lu-FAPI-04, [^90^Y]Y-FAPI-46, [^177^Lu]Lu-FAP-2286, [^177^Lu]Lu-DOTA.SA.FAPI, [^177^Lu]Lu-DOTAGA.(SA.FAPi)_2_, [^177^Lu]Lu-DOTAGA.Glu.(FAPi)_2_, [^177^Lu]Lu-EB-FAPI, and [^213^Bi]Bi-FAPI-46 [19,20,21,22,23,30,35,36,37,38,39,40,41,42,43] (Table 2). Notably, these studies have demonstrated the feasibility and safety of repeated FAP-TRT administrations. Dosimetric analyses have shown that several FAP-targeted radiopharmaceuticals deliver relatively low absorbed doses to the organs at risk (Table 1), supporting an acceptable short-term safety profile in early clinical studies (Table 2). However, the safety profiles appear to be highly compound-dependent. In particular, retention-enhanced constructs, including albumin-binding approaches, may increase the systemic exposure and bone marrow absorbed dose. Therefore, safety interpretation should consider ligand design, whole-body residence time, bone marrow exposure, administered activity, and prior therapies.

Haematotoxicity represents the most frequently observed adverse event, aligning the toxicity profile of FAP-TRT with other established TRTs, namely [^177^Lu]Lu-DOTATATE and [^177^Lu]Lu-PSMA (Table 2). Non-haematologic grade ≥3 adverse events were uncommon, and occurred sporadically across the studies, including transient flare reactions in selected cohorts.

The repeated low-activity administration of [^177^Lu]Lu-FAPI-04 demonstrated excellent tolerability in a small heterogeneous cohort, although a formal RECIST-based response assessment was lacking [35]. Subsequent early-phase experiences with [^177^Lu]Lu-FAPI-46 confirmed favourable safety profiles, with a predominantly stable disease observed in heavily pretreated patients and only occasional grade III haematologic toxicity [36]. While these first-in-human studies have collectively supported the feasibility and safety of repeated FAP-TRT, they have also highlighted limited tumour retention as a potential constraint on achieving objective responses.

To address this limitation, second-generation FAP-targeting radiopharmaceuticals aimed to enhance tumour retention through dimerization or peptide modification. Dimerization strategies, such as [^177^Lu]Lu-DOTAGA.(SA.FAPi)_2_, demonstrated an improved tumour retention time compared to a monomeric tracer [21], laying the groundwork for retention-optimised strategies. Similarly, the cyclic peptide ligand [^177^Lu]Lu-FAP-2286 showed prolonged tumour uptake in heterogeneous advanced malignancies [19], although objective responses remained modest in early cohorts and haematologic toxicity became more evident; transient grade III flare reactions, including tumour-associated pain, were also reported in selected patients. Beyond dimerization and peptide-based strategies, additional ligand-engineering approaches have sought to refine the small-molecule FAPI constructs in order to optimise tumour retention while maintaining favourable clearance kinetics. In a first-in-human phase I dose-escalation study, [^177^Lu]Lu-FAPI-XT (XT117) was evaluated in 14 patients with advanced FAP-positive solid tumours, predominantly soft-tissue sarcomas, including fibroblastic sarcoma, undifferentiated pleomorphic sarcoma, and leiomyosarcoma. Disease stabilisation was observed in 5 of 14 patients (35.7%), and the most favourable signal was in fibroblastic sarcoma subtypes, where stable disease was reported in 4/8 patients (50%), and no grade ≥3 treatment-related haematologic adverse events were reported [37].

Parallel efforts explored the impact of radionuclide selection. The use of higher-energy, longer-range β-emitters, such as ^90^Y with [^90^Y]Y-FAPI-46 in advanced sarcoma and other solid tumours, demonstrated a feasibility and disease control rate of approximately 48% in the most recent cohort, suggesting a modest but encouraging signal of efficacy, but was associated with increased grade III/IV haematologic toxicity [22,23,38]. These findings suggest that, while higher-energy β-emitters may enhance cytotoxicity in sarcomas, where the FAP expression can be present on tumour cells, the increased haematotoxicity underscores the importance of dosimetry optimisation. Beyond β-emission strategies, alpha-emitter therapy has recently entered clinical evaluation. Fractionated [^213^Bi]Bi-FAPI-46 demonstrated preliminary evidence of therapeutic efficacy without significant adverse events in a small pilot study (*n* = 6) [39]. However, the response assessment was based on a visual imaging evaluation rather than standardised RECIST criteria or central independent radiologic review, limiting direct comparability with other studies. Although the patient numbers remain small, alpha-particle FAP-TRT offers theoretical advantages, including high linear energy transfer, which may be particularly beneficial in tumours where the FAP is expressed by the tumour cells themselves.

More encouraging tumour-specific outcomes have emerged in selected settings. In radioiodine-refractory differentiated thyroid cancer, treatment with the dimeric [^177^Lu]Lu-DOTAGA.(SA.FAPi)_2_ resulted in partial responses in a subset of patients without grade III/IV toxicity. Although differentiated thyroid cancer is not generally regarded as a uniformly desmoplastic tumour type, selected radioiodine-refractory cases may show clinically relevant FAPI uptake, indicating the presence of a therapeutically targetable FAP-positive tumour microenvironment [40]. Similarly, in metastatic breast cancer, dimeric FAPI ligands ([^177^Lu]Lu-DOTAGA.(SA.FAPi)_2_ or [^177^Lu]Lu-DOTAGA.Glu.(FAPi)_2_) yielded notable response rates, including five complete responses and three partial responses, corresponding to an objective response rate of 42.1% in heavily pretreated patients. However, the response assessment was based on visual analogue scoring rather than standardised RECIST criteria, limiting direct comparability with other trials. In advanced sarcoma, peptide-based [^177^Lu]Lu-FAP-2286 achieved an objective response rate of 50% in the overall treated cohort, albeit in a small sample (*n* = 5), and with an incomplete response assessment in several patients (*n* = 3). Importantly, no grade III/IV toxicities were reported in that series [41]. Other FAP-TRT approaches have also reported disease control in sarcoma, although outcomes remain heterogeneous across the studies [22,23,38]. Taken together, these tumour-specific findings suggest that clinical benefit may depend less on tumour histology, per se, and more on the presence of a FAP-enriched tumour microenvironment identifiable on molecular imaging.

Among the FAPI derivatives designed to prolong the tumour residence time, albumin-binding approaches have demonstrated promising efficacy and represent one of the most clinically advanced FAP-TRT strategies to date. A dose-escalation evaluation of [^177^Lu]Lu-EB-FAPI (LNC1004) in metastatic radioiodine-refractory thyroid cancer demonstrated promising response signals but also revealed dose-dependent haematologic toxicity, with grade IV thrombocytopenia reported at higher activity levels, illustrating the therapeutic trade-off between prolonged tumour retention and increased systemic radiation exposure inherent to albumin-binding constructs [20]. Building upon these early findings, Fu et al. reported phase II results of [^177^Lu]Lu-EB-FAPI (LNC1004) in 28 patients with pretreated metastatic cancers, predominantly thyroid carcinoma, breast cancer, neuroendocrine carcinoma, and sarcoma. Among the twenty evaluable patients, four achieved partial responses and nine had stable disease cases according to RECIST, corresponding to an objective response rate (ORR) of 20% and a disease control rate (DCR) of 65%. On an intention-to-treat basis (*n* = 28), the ORR and DCR would correspond to 14.3% and 46.4%, respectively. However, grade III–IV haematologic toxicity occurred in six patients (mostly thrombocytopenia), and bone pain flare was observed in seven patients [44], further emphasising that myelosuppression represents the principal dose-limiting toxicity across FAP-TRT.

When placed in context, the ORRs observed across the FAP-TRT applications have generally remained below 20% in heterogeneous, heavily pretreated populations, with higher response rates occasionally observed in selected tumour-specific cohorts. By contrast, established radioligand therapies have demonstrated higher response rates in phase III settings within specific selected tumour types. In the VISION trial, [^177^Lu]Lu-PSMA-617 achieved an ORR of 29.8% in metastatic castration-resistant prostate cancer [45], while, in the NETTER-1 trial, [^177^Lu]Lu-DOTATATE demonstrated an ORR of 18% in advanced midgut neuroendocrine tumours [46]. Importantly, these trials were conducted in tumour-specific populations with molecular selection strategies, whereas current FAP-TRT studies largely follow tumour-agnostic basket designs. Such heterogeneity in tumour biology may dilute the ORRs and obscure signals of activity within particularly FAP-enriched tumour subsets.

Collectively, early-phase clinical data indicate that FAP-TRT is feasible across multiple radiopharmaceuticals and tumour types. Additionally, although the response assessment criteria have varied across the studies, most commonly RECIST, the aggregated data suggest an overall disease control rate of approximately 60–70% in heavily pretreated populations (Table 2). However, efficacy appears strongly influenced by compound design, radionuclide selection, tumour biology, and patient selection strategy, while haematologic toxicity remains the dominant safety concern, particularly for retention-enhanced constructs. These considerations highlight the need for disease-focused prospective trials and refined imaging-based selection criteria to more accurately define the therapeutic potential of FAP-TRT.

Ongoing phase I and II trials (Table 3) are expected to provide more conclusive insights into the efficacy and therapeutic potential of FAP-TRT. However, not all early development programs have progressed successfully. The phase I FRONTIER trial evaluating [^177^Lu]Lu-PNT6555 (NCT05432193) has been listed as terminated after completion of initial cohorts and discontinuation of further expansion, likely due to rapid clearance of the agent resulting in insufficient tumour retention and absorbed tumour dose [47], thereby highlighting the challenges of translating some FAP-targeted constructs into later-phase development.

## 5. Future Directions and Combination Strategies

A unique feature of FAP-TRT is its primary action on CAFs within the tumour stroma, indirectly affecting tumour cells through structural and functional changes in the TME. This opens opportunities for synergistic combinations with systemic therapies. Certain studies have demonstrated that FAP-expressing CAFs play a key role in promoting an immunosuppressive TME and in mediating resistance to immune checkpoint inhibitors (ICIs). In colorectal cancer models, high FAP expression in CAFs was shown to drive immunosuppression through increased CCL2 secretion, myeloid cell recruitment, and T-cell inactivation, ultimately contributing to ICI resistance [48]. Similarly, findings in gastric cancer revealed that high FAP expression in CAFs actively promotes tumour progression, immunosuppression, and drug resistance. Notably, inhibition of the FAP in CAFs was shown to sensitise gastric cancer to immune checkpoint blockade, suggesting that targeting the FAP may restore immune responsiveness and enhance the efficacy of immunotherapies across multiple tumour types [49]. These mechanistic observations provide a strong biological rationale for combining FAP-TRT with immune checkpoint inhibition, particularly in tumours characterised by immune exclusion. Preclinical studies have demonstrated highly promising synergistic effects when FAP-TRT is combined with ICIs, such as anti-PD-1 or anti-PD-L1 antibodies. Treatment with [^177^Lu]Lu-DOTA-EB-FAPI (LNC1004) in combination with PD-L1 inhibition induced durable tumour regression and immunological memory upon rechallenge, accompanied by increased CD8^+^ T-cell infiltration and reprogramming of the tumour microenvironment [50]. Similarly, [^177^Lu]Lu-FAP-2287 enhanced anti-PD-1-mediated tumour control through activation of type I interferon signalling, increased cytotoxic T-cell recruitment, and reduction of immunosuppressive myeloid cell populations [51]. An additional study using [^177^Lu]Lu-DOTA-2P(FAPI)_2_ further demonstrated enhanced tumour immunogenicity and improved responsiveness to PD-L1 blockade [52]. These convergent findings suggest that FAP-TRT may function not only as a stromal-targeted cytotoxic modality but as an immune-priming strategy capable of reshaping CAF-driven immune exclusion. By increasing tumour antigenicity and facilitating effector T-cell infiltration, FAP-TRT may help convert immunologically “cold” tumours into inflamed “hot” phenotypes more susceptible to checkpoint inhibition, thereby providing a strong rationale for prospective clinical combination trials [7,53,54]. Future clinical translation will require careful evaluation of sequencing strategies (priming versus concurrent administration) and monitoring for potential overlapping inflammatory toxicities [7].

Beyond immune checkpoint inhibition, additional combination strategies aim to enhance cytotoxic efficacy. A study by Bao et al. demonstrated that co-treatment with PARP inhibitors can significantly enhance the therapeutic efficacy of FAP-TRT. The synergistic efficacy observed with the addition of Olaparib underscores the potential of targeting DNA damage repair pathways to augment the therapeutic effects of FAP-TRT. Inhibiting PARP-mediated repair enhances radiation-induced cytotoxicity, providing a rationale for further exploration of DNA damage response inhibitors in combination with FAP-TRT [55].

Combination strategies with cytotoxic chemotherapy are also biologically justified. FAP-expressing CAFs contribute to extracellular matrix deposition, increased interstitial pressure, and impaired drug penetration, thereby mediating chemoresistance in desmoplastic tumours, such as pancreatic ductal adenocarcinoma and breast cancer [56,57,58]. By selectively targeting and irradiating FAP-positive stromal elements, FAP-TRT may disrupt this protective fibrotic barrier, potentially enhancing the intratumoural delivery and efficacy of systemic chemotherapy. Moreover, radiation-induced DNA damage from [^177^Lu]Lu-based therapy may exert additive or synergistic effects with the cytotoxic agents that impair DNA replication or repair, analogous to the radiosensitisation effects observed with PARP inhibition. Early-phase clinical development programs, such as the LuMIERE trial (NCT04939610), have therefore incorporated chemotherapy-containing cohorts to explore whether stromal modulation by [^177^Lu]Lu-FAP-2286 can improve therapeutic outcomes in heavily pretreated solid tumours.

However, given that haematologic toxicity represents the principal dose-limiting adverse event of FAP-TRT, careful dose optimisation and bone marrow monitoring are essential when combining with myelosuppressive chemotherapy, radiosensitisers, or DNA damage response inhibitors. These findings collectively highlight the growing momentum behind combinatorial approaches that aim to potentiate FAP-TRT efficacy and broaden its therapeutic impact across various tumour models.

In conclusion, FAP-TRT represents a promising and innovative therapeutic approach in nuclear oncology, with the potential for broad applicability across various tumour types. Continued advancements in FAP-targeted radiopharmaceutical development, alongside a deeper understanding of the molecular and cellular mechanisms underlying FAP-TRT and the combination strategies with other anti-cancer treatments will be pivotal in optimising its clinical utility and therapeutic impact. Future tumour-specific prospective trials incorporating imaging-based selection and robust toxicity and response monitoring will be essential to accurately define the role of FAP-TRT within the evolving theranostic landscape. 

## Figures and Tables

**Table 1:** Comparison of the effective half-lives and absorbed doses of FAP-targeted therapeutic radiopharmaceutical with [^177^Lu]Lu-PSMA-617 and [^177^Lu]Lu-DOTATATE.

Radiopharmaceutical	Whole Body Half-Life (h)	Tumour Half-Life (h)	Kidney Half-Life (h)	Tumour Absorbed Dose	Bone Marrow Dose	Kidney Dose
[^177^Lu]Lu-DOTATATE	21–161 (range) [16]	99 (median, all lesions) [15]	52 (median) [17]	2–6 Gy/GBq (mean) [18]	0.02–0.05 Gy/GBq (mean) [18]	0.4–0.9 Gy/GBq (mean) [18]
[^177^Lu]Lu-PSMA-617 [14]	40 (mean)	54 (mean, all lesions) 74 (mean, bone lesions)	42 (mean)	5.3 Gy/GBq (mean, all lesions) 2.9 Gy/GBq (mean, bone lesions)	0.03 Gy/GBq (mean)	0.8 Gy/GBq (mean)
[^177^Lu]Lu-FAP-2286 [19]	35 (mean)	44 (mean, bone lesions)	81 (mean)	3 Gy/GBq (mean, bone lesions)	0.05 Gy/GBq (mean)	1 Gy/GBq (mean)
[^177^Lu]Lu-EB-FAPI (LNC1004) [20]	90 (mean)	92 (mean, all lesions) 99 (mean, bone lesions)	101 (mean)	8.50 Gy/GBq (mean, all lesions) 5.12 Gy/GBq (mean, bone lesions)	0.11 mSv/MBq (mean)	1.32 mSv/MBq (mean)
[^177^Lu]Lu-DOTAGA.(SA.FAPi)_2_ [21]	46.2 (median)	86.6 (median, all lesions)	NA	6.7 Gy/GBq (median, all lesions) 6.07 Gy/GBq (median, bone lesions)	0.017 Gy/GBq (mean)	0.37 Gy/GBq (mean)
[^90^Y]Y-FAPI-46 [22,23]	NA	8.7 (median, all lesions)	NA	2.81 Gy/GBq (mean, highest uptake lesion)	0.04 Gy/GBq (mean)	0.53 Gy/GBq (mean)

**Table 2:** Clinical Studies of FAP-targeted Radioligand Therapy.

Author and Publication Year	N (Treated)	Tumour Type	FAP-Targeting Agent	Treatment Cycles	Median/ Mean Injected Activity	Response	Grade ≥ 3 Haematologic Adverse Events (N)
Lanzafame et al., 2026 [38]	30	Sarcoma (*n* = 23) and other solid tumours (pancreatic cancer, prostate cancer, gastric cancer, nonmelanoma skin cancer, and cholangiocarcinoma)	[^90^Y]Y-FAPI-46	1–6	3.7–7.4 GBq	3PR, 9 SD (RECIST)	8
Liu et al., 2026 [37]	14	Sarcomas, CRC, thyroid, and NSCLC	[^177^Lu]Lu-FAPI-XT (XT117)	1–6	3.7–11.1 GBq	5 SD (RECIST)	0
Fu et al., 2025 [44]	28	Various tumours with high FAP expression (Thyroid, BC, NEC, sarcoma, NSCLC, gastroesophageal cancer, CRC, nasopharyngeal, renal cancers)	[^177^Lu]Lu-EB-FAPI (LNC1004)	2–4	3.3 GBq	4 PR, 9 SD 8 NA (RECIST)	6
Wang et al., 2025 [42]	4	Solitary fibrous tumour	[^177^Lu]Lu-EB-FAPI	1	2.22 GBq	3 SD (PERCIST/RECIST)	0
Helisch et al., 2024 [39]	6	Colon, anal, BC, prostate cancer	[^213^Bi]Bi-FAPI-46	5–12	1.609 MBq	1 PR, 1 SD, 4 PD (visual assessment)	0
Banihashemian et al., 2024 [41]	8	Sarcoma	[^177^Lu]Lu-FAP-2286	2–4	6.6 GBq, −7.4 GBq	4 PR, 1 PD, 3 NA (RECIST)	0 in evaluable patients
Yadav et al., 2024 [43]	19	BC	[^177^Lu]Lu-DOTAGA.(SA.FAPi)2 and [^177^Lu]Lu-DOTAGA.Glu.(FAPi)2	2–6	5.5 GBq	5 CR, 3 PR, 8 minimal response, 2 SD, 1 no response (visual analog score)	0
Fu et al., 2023 [20]	12	Thyroid cancer	[^177^Lu]Lu-DOTA-EB-FAPI (LNC1004)	2	2.28 GBq, −4.80 GBq	3 PR, 7 SD, 2 PD (RECIST)	3
Fendler et al., 2022 [22]	21	Sarcoma, PC, prostate, gastric cancer	[^90^Y]Y-FAPI-46	1–4	3.7–7.4 GBq	1 PR, 7 SD, 8 PD (RECIST)	4
Assadi et al., 2021 [36]	18	OC, sarcoma, CRC, BC	[^177^Lu]Lu-FAPI-46	1–4	3.7 GBq (1.85–4.4 GBq)	12 SD, 6 PD (RECIST)	1
Ballal et al., 2022 [40]	15	Thyroid cancer	[^177^Lu]Lu-DOTAGA.(SA.FAPI)2	2–4	8.2 GBq (5.5–14 GBq)	4 PR, 3 SD (molecular response assessment)	0
Baum et al., 2022 [19]	11	PC, BC, OC, rectum	[^177^Lu]Lu-FAP-2286	1–3	5.8 GBq (2.4–9.9 GBq)	2 SD, 9 PD (RECIST)	2
Ferdinandus et al. 2022 [23]	9	Sarcoma, PC	[^90^Y]Y-FAPI-46	1–3	3.8 GBq (IQR 3.25–5.4 GBq) for the first cycle, 7.4 GBq (IQR 7.3–7.5) for any subsequent cycle	4 SD, 4 PD (RECIST)	4
Ballal et al., 2021 [21]	7	Thyroid, paraganglioma	[^177^Lu]Lu-DOTAGA.(SA.FAPI)2	3 (median)	1.48 GBq (IQR: 0.6–1.5)	NA	1
Kuyumcu et al., 2021 [35]	4	BC, thymic, thyroid, OC	[^177^Lu]Lu-FAPI-04	1	0.27 GBq (0.26–0.28 GBq)	NA	0

**Table 3:** Ongoing Studies in FAP-targeted Theranostics.

ClinicalTrials.gov ID and Status of the Study	Study Title	Phase	Estimated Enrolment	Tumour Type	Intervention/Treatment	Primary Endpoint
NCT04939610 (recruiting)	LuMIERE: A Phase 1/2, Multicenter, Open-label, Non-randomized Study to Investigate Safety and Tolerability, Pharmacokinetics, Dosimetry, and Preliminary Activity of [^177^Lu]Lu-FAP-2286 in Patients With an Advanced Solid Tumour	I/II	Phase 1: 30 Phase 2: 192	Phase 1: FAP expressing solid tumours. Phase 2: PDAC, and NSCLC (monotherapy and combination with Cht), BC (monotherapy only)	[^68^Ga]Ga-FAP-2286 (as imaging agent) [^177^Lu]Lu-FAP-2286 (as therapeutic agent)	DLTs, RP2D, (s)AEs, ORR
NCT05400967 (recruiting)	Diagnosis of Metastatic Tumours on [^68^Ga]Ga-FAPI PET-CT and Radioligand Therapy with [^177^Lu]Lu-EB-FAPI	I	10	Treated or untreated metastatic solid tumours	[^68^Ga]Ga-FAPI (as imaging agent) and 177Lu-EB-FAPI (as therapeutic agent)	(s)AEs, therapeutic response assessed by [^68^Ga]Ga-FAPI PET/CT
NCT06638034 (recruiting)	Diagnosis of Metastatic Tumours on [^68^Ga]Ga-FAPI-RGD PET-CT and Radioligand Therapy	I	15	FAP-Expressing Positive Tumours RGD-Expressing Positive Tumours	[^68^Ga]Ga-FAPI (as imaging agent) and [^177^Lu]Lu-FAPI-RGD (as therapeutic agent)	Uptake value of [^177^Lu]Lu-FAPI-RGD in normal organs and tumours, (s)AEs
NCT05723640 (completed)	Phase I, Open-Label Study of the Safety and Dosimetry of a 4-Dose Regimen of Escalating Doses of [^177^Lu]Lu-LNC1004 Injection in Adult Patients With Advanced Fibroblast Activation Protein (FAP)-Positive Solid Tumours	I	24	Treated solid tumours	[^177^Lu]Lu-FAPI-46-EB (LNC1004)	(s)AEs, DLTs, MTD
NCT04849247 (unknown status)	[^68^Ga]Ga-DOTA-FAPI and [^177^Lu]Lu-DOTA-FAPI Theranostic Pair in Patients With Various Types of Cancer (Locally Advanced or Metastatic Cancer)	I	30	Treated locally advanced or metastatic cancer	[^68^Ga]Ga-FAPI (as imaging agent) and [^177^Lu]Lu-DOTA-FAPI (as therapeutic agent)	Assessment of [^68^Ga]Ga-FAPI PET/CT imaging to detect lesions, DLT
NCT06081322 (recruiting)	An Exploratory Study to Evaluate the Safety and Efficacy of Peptide Receptor Radionuclide Therapy With [^177^Lu]Lu-EB-FAPI in Patients With Advanced Cholopancreatic Tumours	I	29	Treated advanced PDAC and cholangiocarcinoma	[^177^Lu]Lu-EB-FAPI	(s)AEs, ORR
NCT06710756 (recruiting)	Lead-212 PSV359 Therapy for Patients With Solid Tumours	I/IIa	112	FAP-expressing solid tumours	[^203^Pb]Pb-PSV359 (as imaging agent) [^212^Pb]Pb-PSV359 (as therapeutic agent)	RP2D, (s)AEs
NCT06636617 (recruiting)	A Phase I Study to Evaluate the Safety, Dosimetry, and Preliminary Efficacy of [^177^Lu]Lu-JH04 in Patients With Advanced FAP-Positive Solid Tumours	I	30	Advanced FAP-positive solid tumours	[^177^Lu]Lu-JH04	DLTs, (s)AEs, dosimetry, RP2D
NCT07213791 (recruiting)	A Study of LY4337713 in Participants With Advanced FAP-Positive Solid Tumours (FiREBOLT)	I	60	Advanced FAP-positive solid tumours	LY4337713	(s)AEs, DLTs, MTD/RP2D, pharmacokinetics
NCT07156565 (Not yet recruiting)	Actinium Therapy for Late-stage Aggressive Sarcomas (ATLAS)	I/II	26	FAP-positive soft tissue sarcoma	[^64^Cu]Cu-LNTH-1363S (as imaging agent) [^225^Ac]Ac-RTX-2358 (as therapeutic agent)	DLT, MTD/RP2D,(s)AEs
NCT06880757 (Not yet recruiting)	[^177^Lu]Lu-FAP-2286 Treatment in Urothelial Neoplasms: Utility and Safety as a Novel Treatment. (FAUNUS)	II	10	Refractory urothelial bladder cancer	[^177^Lu]Lu-FAP-2286	ORR, dosimetry, (s)AEs

Clinical trial information was retrieved from ClinicalTrials.gov, with the search performed on 23 February 2026.

## Data Availability

No new data were created or analyzed in this study. Data sharing is not applicable to this article.

## Abbreviations

The following abbreviations are used in this manuscript:

AE/AEs	adverse event(s)
BC	breast cancer
CAF/CAFs	cancer-associated fibroblast(s)
CRC	colorectal cancer
Cht	chemotherapy
CR	complete response
DCR	disease control rate
DLT/DLTs	dose-limiting toxicity/toxicities
DNA	deoxyribonucleic acid
DOTA	1,4,7,10-tetraazacyclododecane-1,4,7,10-tetraacetic acid
EB	Evans blue
FAP	fibroblast activation protein
FAP-TRT	FAP-targeted radionuclide therapy
FAPI	fibroblast activation protein inhibitor
G	grade
GBq	gigabecquerel
Gy	gray
h	hours
ICI/ICIs	immune checkpoint inhibitor(s)
ITT	intention-to-treat
IQR	interquartile range
mAb	monoclonal antibody
MBq	megabecquerel
mGy	milligray
mSv	millisievert
MTD	maximum tolerated dose
N	number of patients
NA	not available
NEC	neuroendocrine carcinoma
NSCLC	non-small cell lung cancer
OC	ovarian cancer
ORR	objective response rate
p	patient(s)
PARP	poly(ADP-ribose) polymerase
PC	pancreatic cancer
PD	progressive disease
PD-1	programmed cell death protein 1
PD-L1	programmed death-ligand 1
PDAC	pancreatic ductal adenocarcinoma
PERCIST	PET Response Criteria in Solid Tumours
PET	positron emission tomography
PET/CT	positron emission tomography/computed tomography
PR	partial response
PRRT	peptide receptor radionuclide therapy
PSMA	prostate-specific membrane antigen
RECIST	Response Evaluation Criteria in Solid Tumours
RGD	arginine-glycine-aspartic acid
RP2D	recommended phase 2 dose
(s)AEs	serious adverse events
SD	stable disease
SPECT	single-photon emission computed tomography
SPECT/CT	single-photon emission computed tomography/computed tomography
SSTR	somatostatin receptor
TME	tumour microenvironment
TRT/TRTs	targeted radionuclide therapy/therapies
